# Physical activity among transgender individuals: A systematic review of quantitative and qualitative studies

**DOI:** 10.1371/journal.pone.0297571

**Published:** 2024-02-28

**Authors:** Joseph S. Lightner, Justin Schneider, Amanda Grimes, Melissa Wigginton, Laurel Curran, Tori Gleason, Tyler Prochnow

**Affiliations:** 1 School of Nursing and Health Studies, University of Missouri-Kansas City, Kansas City, Missouri, United States of America; 2 American Public Health Association, Physical Activity Section, Washington, D.C., United States of America; 3 Department of Nursing, California State University-San Bernardino, Palm Desert, California, United States of America; 4 Department of Health Behavior, School of Public Health, Texas A&M University, College Station, Texas, United States of America; 5 University of Kansas Medical Centers, Kansas City, Kansas, United States of America; The Hong Kong Polytechnic University, HONG KONG

## Abstract

Transgender individuals face stigma, discrimination, and other barriers impacting their ability to engage in physical activity (PA). We aim to review current literature on PA among transgender individuals. A systemic literature search of research studies from 2010–2023 was conducted. Studies must have reported a measure of PA and gender, be original research, and focus on transgender participants’ PA. Rates of PA for transgender individuals were lower compared to cisgender or sexual minority individuals. Transgender women were less likely to engage in PA than other groups. Qualitative results suggest transgender oppression, stigma, discrimination, body image, unwelcoming environments (gyms, locker rooms, swimming pools), and the dichotomous structure of sport contribute to lower rates of PA among transgendered individuals. Disparities in PA for transgender individuals exist. Policy, environment, and system changes are needed to reduce transgender stigma in sport and PA settings. Current legislation is being developed and implemented in the United States regarding the place of transgender individuals in sport and PA. These results should inform public discourse on the topic.

## Introduction

Physical activity (PA) is a fundamental aspect of health and well-being, with numerous physical, mental, and social benefits [1]. However, transgender individuals face unique challenges that may impact their ability to engage in PA which can contribute to health disparities and worse health outcomes [2]. Transgender individuals are those whose gender identity differs from the sex assigned at birth, and they may experience stigma, discrimination, and barriers to accessing healthcare and other services [3].

These challenges may also extend to their ability to engage in PA, including sports, fitness, and recreational activities [2]. These individuals may identify outside of the male/female binary gender construct and identify as nonbinary, which can exist anywhere on the gender spectrum or can indicate no having a gender at all [4]. They report well-founded fear of sport and physical education (PE) spaces due previous negative interactions and experience with transphobia, harassment, and exclusion [5–8]. In this study, we use the term transgender and gender diverse (TGD) to refer to the wide array of gender expressions. This is consistent with the eighth edition of the World Professional Association for Transgender Health (WPATH) Standards of Care [4].

The health disparities experienced by transgender individuals are well-documented and include higher rates of mental health problems, cardiovascular disease, and cancer [9]. Lack of PA is also a significant contributor to these health disparities, as PA decreases the risk of obesity, diabetes, and other chronic conditions [1]. Transgender individuals may face additional barriers to PA due to concerns about their safety, privacy, and access to gender-affirming facilities [2, 3, 9].

The current literature on PA among transgender individuals is limited, with few studies exploring their experiences, barriers, and facilitators to PA [2]. However, existing studies suggest that transgender individuals may face unique challenges related to PA, including discrimination, harassment, and exclusion from sports and fitness programs [2]. Transgender individuals may also face challenges related to body dysphoria, which can impact their ability to feel comfortable and confident in their bodies during PA [10]. The systematic exclusion of transgender individuals in physical activity research has led to a call to action to improve the field’s understanding of these unique challenges on physical activity participation [11].

In this article, we aim to review the current literature on PA among transgender individuals, including the rate of PA, barriers and facilitators to engagement in PA, and the potential impact on health outcomes. We will explore the ways in which transgender individuals experience PA, including the impact of gender identity on participation in sports, fitness, and other physical activities. We will also discuss the implications for future research and policy initiatives related to PA among transgender individuals. Within this discussion we will center the potential for policy initiatives that promote inclusion and equality in sports and fitness programs as well as the need for gender-affirming facilities that can meet the specific needs of transgender individuals.

## Materials and methods

### Search strategy

A search of peer-reviewed research studies from January 1, 2010- January 24, 2023 was conducted. Web of Science, APA PsycInfo, CINAHL Complete, Pub Med, EBSCO, and Academic Search Ultimate were searched to identify relevant English language studies. Keywords for all database searches were: (“physical activit*” OR “exercis*” OR “workout” OR “active” OR “active play” OR “aerobic” OR “inactivity” OR “walking” OR “active transport” OR “physical education*” OR “sedentary” OR “sport”) AND (“transgender” OR “gender non-conforming” OR “gender creative” OR “gender incongruent” OR “gender diverse” OR “gender non-binary” OR “trans and nonbinary” OR “gender nonbinary” OR “transexual” OR “transsexual” OR “transwoman” OR “transman” OR “trans woman” OR “trans man”).

### Selection criteria

**Fig 1:**
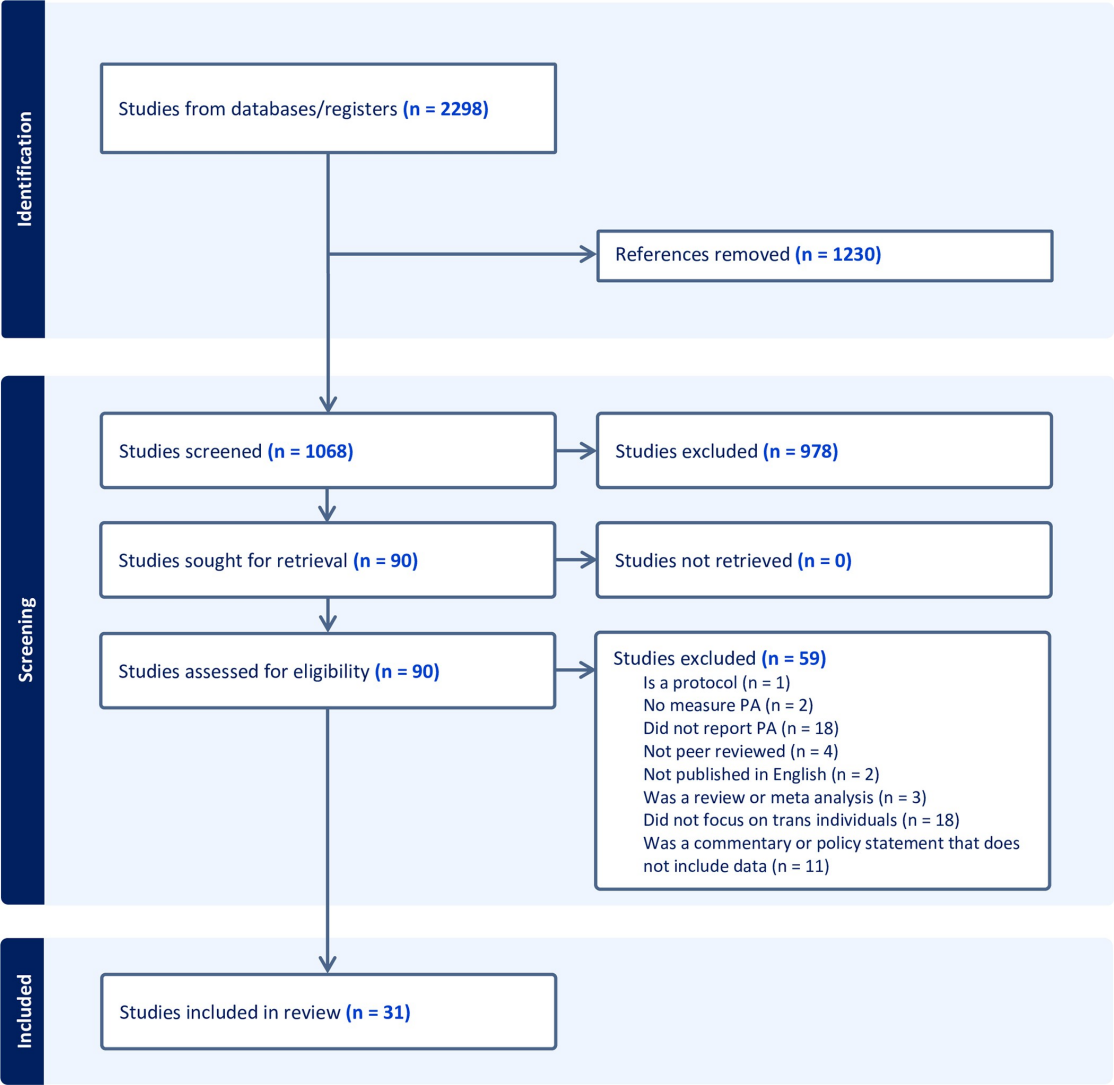
PRISMA diagram here.

To be included in the review, studies must have been published between 2010 and 2023, report a measure of PA and gender, be original research, and independently report variables related to PA for transgender individuals. Studies were excluded if they reported variables related to PA in aggregate with other groups (e.g., with gay, lesbian, and bisexual individuals). All abstracts (*n =* 1,068) were independently reviewed by two investigators. Full-text review (*n =* 90) was conducted by two independent investigators. When discrepancy between the two reviewers existed, the first and senior authors made the determination to include or exclude the article from the study. Inter-rater reliability among investigators was excellent (Cohen’s Kappa = 0.77). The PRISMA checklist is presented in S1 Checklist. See Fig 1 for the PRISMA diagram.

### Data extraction and appraisal

Authors discussed and agreed upon an extraction and appraisal strategy. Initial extraction was conducted by one researcher. Data elements included: title, year, country, study aims, study design, measures of PA and gender, primary and secondary outcomes, and implications. Articles were appraised using and adapted checklist (See Table 1) containing 15 criteria common to observational studies [12, 13]. Criteria had a yes (1 point); no (0 points); or unclear (0 points) answer format. All criteria had the same weight and a quality score ranging from 0 to 15 points was calculated for each study. The average article quality from appraisals was 8.77 points out of 15, SD = 2.88. Data elements and appraisals were checked by an additional researcher. All authors contributed to review, extraction, and appraisal. Two tables were developed to summarize author, year, dataset or measure of physical activity, study population, sample, and a summary of primary and secondary outcomes.

**Table 1:** Criteria for quality assessment and the number (%) of studies scoring a point for each separate item.

Criterion	Description	*n* (%)
Objectives	Are the objectives or hypotheses of the research described in the paper stated?	28 (90)
Study design	Is the study design presented?	30 (97)
Target population	Do the authors describe the *target* population they wanted to research?	29 (94)
Sample	Was a random sample of the target population taken? AND was the response rate 60% or more?	1 (3)
Sample	Is participant selection described?	24 (77)
Sample	Is participant recruitment described, or referred to?	25 (81)
Sample	Are the inclusion and/or exclusion criteria stated?	21 (68)
Sample	Is the study sample described? (minimum description *=* sample size, gender, age and an indicator of SES)	17 (55)
Sample	Are the numbers of participants at each stage of the study reported? (Authors should report at least numbers eligible, numbers recruited, numbers with data at baseline, and numbers lost to follow-up)	15 (48)
Variables	Are the measures of physical activity and gender described?	19 (61)
Data sources and collection	Do authors describe the source of their data (e.g., cancer registry, health survey) AND did authors describe how the data were collected? (e.g., by mail)	30 (97)
Measurement	Was reliability of the measure(s) of physical activity mentioned or referred to?	4 (13)
Measurement	Was the validity of the measure(s) of physical activity mentioned or referred to?	2 (6)
Statistical methods	Were appropriate statistical methods used and described, including those for addressing confounders?	21 (68)
Statistical methods	Were the numbers/percentages of participants with missing data for physical activity indicated AND If more than 20% of data in the primary analyses were missing, were methods used to address missing data?	6 (19)

## Results

A total of 31 articles met the inclusion criteria, 15 quantitative and 16 qualitative. Most studies were conducted in the United States (*n =* 17) with additional studies conducted in the United Kingdom (*n =* 5), Brazil (*n =* 2), Germany (*n =* 1), Canada (*n =* 1), Finland (*n =* 1), the Netherlands (*n =* 1), Australia (*n =* 1), Spain (*n =* 1), and Ireland (*n =* 1). Total sample sizes ranged from 2 to 358,543, with the largest sample of transgender individuals being 2,070. Surveillance data from the Behavioral Risk Factor Surveillance System, Youth Risk Behavior Survey, National College Health Assessment, Minnesota Student Survey, and California Healthy Kids Survey was used in eight studies. Transgender youth were a particular focus in five quantitative studies.

### Quantitative results

Table 2 presents the results of quantitative studies. In general, across all ages, transgender individuals engage in less PA than cisgender individuals. In youth (grades 9–12), Voss, et al. [14] reported that among a national sample in the United States, transgender youth were less than half as likely to participate in PA as cisgender peers. Additionally, trans women were less likely to participate in sport. Among 8^th^-11^th^ graders in Minnesota, Espinoza et al. [15] and Bishop et al. [16] reported transgender youth were less likely to participate in team sports, PA lessons, and achieved less PA than cis-gender youth. Pistella, et al. [17] did not identify a difference in PA among youth in California. However, transgender youth in California reported feeling less safe at school and engaging in less physical education than cisgender youth.

**Table 2:** Summary of quantitative studies included in review.

Author and Year	Dataset or PA Measure	Study Population	Sample	Outcomes
Bishop et al., 2020	2016 Minnesota Student Survey	8th, 9th, 11th graders in Minnesota	Total = 80,794 Trans men = 1,412 Trans women = 626	More transgender and gender nonconforming students assigned female at birth did not participate in any sports during the week, and more transgender and gender nonconforming students assigned male at birth re-ported 0 days in the last week of being physically active (for at least 60 min/day) compared with all other gender identity subgroups.
Cunningham et al., 2018	2016 Behavioral Risk Factor Surveillance System	U.S. adults (ages 21–65+) from 25 states and Guam	Total = 200,874 Trans men = 234 Trans women = 344	The prevalence of performing any leisure-time exercise was higher among cisgender adults (75.5%) than among male-to-female transgender adults (56.7%). 69.0% of female-to-male transgender adults reported engaging in physical activity (PA) in the last 30 days.
Espinoza et al., 2023	Minnesota Student Survey	8th, 9th, 11th graders in Minnesota	Total = 125,375 Trans men = 625 Trans women = 203	Team sport and PA lesson participation were significantly associated with gender modality. Approximately one in four transgender and gender diverse adolescents participated in a sport, compared to a third of questioning and more than half of cisgender adolescents. PA also differed significantly by gender modality; transgender and gender diverse adolescents had the lowest PA (avg trans women = 2.72 days/week; trans men = 2.52 days/week), and cisgender adolescents had the highest PA (avg female = 3.37 days/week, male = 3.64 days/week).
Fredriksen-Goldsen et al., 2014	Being physically inactive was defined as not being engaged, on a weekly basis, in at least moderate activities that cause some increase in breathing or heart rate	LGBT adults aged 50 and older (mean age = 66.47 years, SD = 9.08 years) in the U.S.	Total = 2,201 Trans men = 61 Trans women = 113	Gender identity effect on physical activity is significant. Transgender older adults were less likely to engage in regular physical activity than non-transgender older adults. The lowered levels of physical activities were linked with poor physical and mental health among transgender older adults.
Hartmann-Tews et al., 2022	Whether they had participated in sports in the previous 12 months (options: yes or no)	LGBTQ+ individuals 16–74 years (mean age = 33.1 years, SD = 11.7 years), living in Germany	Total = 858 Trans men = 44 Trans women = 30	Organized high-performance, and competitive sports may increase the perception of homo-/transnegative language but not negative experiences. Higher prevalence of gay compared to lesbian athletes report negative perception of home-/transnegative language in their sports. Gay athletes and TGD athletes report homo-/transnegative experiences in sport compared to lesbian and cisgender athletes.
Jones et al., 2018	Rapid Assessment of Physical Activity	Transgender individuals aged 17 or older (mean age = 30.15 years, SD = 11.87 years) in the United Kingdom from 2015–2016.	Total = 674 Transgender = 360	Cisgender individuals engaged in significantly more physical activity than transgender individuals. Cisgender men engaged in significantly more physical activity in comparison to trans men. Cisgender women engaged in significantly more physical activity than trans women. Transgender patients who were receiving trans-specific healthcare engaged in significantly more physical activity compared to the patients who were not receiving care.
Muchicko et al., 2014	Godin Leisure Time Exercise Questionnaire	U.S. adults (transgender individuals mean age = 41 years, SD = 2.5 years; cisgender individuals mean age 27 years, SD = 2.0 years).	Total = 80 Trans men = 8 Trans women = 25	Transgender individuals reported lower amounts of leisure time physical activity compared to cisgender individuals and lower social support for physical activity, lower self-efficacy for physical activity, higher peer victimization during childhood.
Nagata et al., 2020	PRIDE Study	U.S. gender and sexual minority adults (transgender men mean age = 30.5 years, SD = 9.7 years, transgender women 41.2 years, SD = 14.9 years)	Total = 484 Trans men = 312 Trans women = 172	Transgender men and transgender women reported lower rates of excessive exercise compared to cisgender men and women.
Pistella et al., 2020	2013–2015 California Healthy Kids Survey	Middle and high school students (grades 6–12) in California schools	Total = 31,609 Transgender = 358	No differences in physical activity were identified for transgender vs cisgender students. Transgender students reported feeling less safe at school and less physical education in school compared to cisgender students.
Silva et al., 2021	Active individuals were those engaged in moderate-intensity physical activity for at least 150 min or in vigorous-intensity physical activity for 75 min per week. Individuals not meeting these criteria were considered sedentary.	Transgender adults (mean age = 32.5 years, SD = 9.0 years) recruited from an outpatient endocrine clinic in Brazil.	Total = 113 Trans men = 53 Trans women = 60	Being physically active was associated with higher physical, psychological, social relations, and environment domain scores when compared to being sedentary. Being physically active was not associated with higher quality of life or sub scales for trans women.
Smalley et al., 2016	Health Risk Questionnaire	U.S. gender and sexual minority adults (mean age = 29.8, SD = 11.4)	Total = 3,279 Trans men = 126 Trans women = 82	24.3% of trans women reported exercising at least 3 days/week, compared to 36.9% of trans men, 35.6% of lesbian women, 38.3% of gay men, and 41.0% of genderqueer or non-binary adults.
VanKim et al., 2014	2007–2011 College Student Health Survey	College students in the U.S. (age ranges 18–25+ years)	Total = 34,271 Transgender = 53	Transgender individuals were less likely to meet recommendations for strenuous physical activity, strengthening physical activity, and screen time.
Voss et al., 2022	2017 and 2019 Youth Risk Behavior Survey	U.S. youth in grades 9–12 (ages 14 and younger to 18+ years)	Total = 156,369 Trans men = 1,054 Trans women = 1,016	Trans men and trans women reported lower odds of physical activity but similar odds of physical education participation compared to cisgendered peers. Trans women were less likely to participate in sports; however, this relationship was not seen in adjusted models. Adjusting for demographics, trans men were significantly more likely to participate in sports. Adjusting for bullying experiences did not significantly change these results.
Wilson et al., 2021	2015–2018 American College Health Association National College Health Assessment	U.S. college students (mean age = 20.0 years, SD = 1.5 years)	Total = 358,543 Trans men = 397 Trans women = 117	Trans men were less likely to meet aerobic and muscle-strengthening activity recommendations compared to cisgender men. No difference was seen between transwomen and cisgender women for aerobic or muscle-strengthening activity.

Note: SD = standard deviation, PA = physical activity.

College-aged transgender individuals reported less PA than cisgender college-aged individuals. Muchicko et al. [18] reported that transgender individuals engaged in less PA and had less social support for PA than cisgender peers. VanKim, et al. [19] reported that transgender college students were less likely to meet PA recommendations and more likely to engage in increased screentime than cis-gender peers. Wilson et. al. [20] reported that transmasculine college students were less likely to meet both aerobic and muscle-strengthening PA recommendations compared to cisgender college students nationally. No differences were seen for transfeminine college students.

Using United States nationally representative data from the Behavioral Risk Factor Surveillance System, Cunningham, et al. [21] reported that adult trans women were less likely to engage in any leisure-time PA compared to cisgender adults. No differences were seen for trans men. In the United Kingdom, Jones, et al. [22] reported that trans men and trans women engaged in less PA than cisgender men and women. Additionally, transgender patients receiving trans-specific healthcare engaged in more PA than transgender patients not receiving trans-specific healthcare. PA in transgender individuals is associated with higher physical and psychological health and better social relationships [23]. Older transgender adults are also less likely to engage in PA than older cis-gender adults [24]. In this study, lower levels of PA in older transgender adults were associated with poorer physical and mental health.

Within lesbian, gay, bisexual, transgender (LGBT+) groups, Smalley et al. [25] reported that trans women were least likely to engage in PA (24.3%), followed by lesbian women (35.6%), trans men (36.9%), and gay men (38.3%). Genderqueer or non-binary adults were the most likely LGBT+ group to engage in PA (41.0%). Transgender adults were less likely to engage in excessive exercise compared to cisgender adults. [26]. Additionally, G**ay and TGD athletes were more likely to report homonegative and transnegative experiences than lesbian or cisgender athletes [27].**

### Qualitative results

Themes identified in the 16 qualitative studies include sport in the male/female binary, body image, exclusion and discrimination, and inclusionary practices. Within exclusion and discrimination, a sub-theme of exclusionary spaces revealed which environments are particularly problematic. The main outcome from each study supports these themes obtained from transgender narratives documented in interviews, focus groups, and surveys. Table 3 provides an overview of qualitative studies included in this review.

**Table 3:** Summary of qualitative studies included in review.

Author and Year	Dataset or PA Measure	Study Population	Sample	Outcomes
Ayala et al., 2020	Mixed methods: open-ended questions and Likert-Style questions related to experiences that affect participation in competitive cycling	Women and gender diverse (WGD) athletes (mean age = 35.94, SD = 9.95)	Total *n =* 386 Transgender and Gender Diverse (TGD) = 27	Themes included representation and community leadership; participants indicated mentoring other WGD athletes, leading clinics, and hosting events were ways to show community support.
Berg &Kokkonen 2022	Interviews related to discrimination in physical education (PE) and sport	LGBTIQ+ students in Finland (ages 13 to 17, mean = 15.7)	Total *n =* 10 TGD = 8	Students had varied experiences with discrimination (from no experience to social exclusion and physical harassment). Half of participants witnessed discrimination in PE; students reported negative locker room experiences. Transgender participants describe problematic relationship with their body making some activities, wearing a t-shirt, and locker rooms uncomfortable; use of chest-binders made breathing difficult during activity. Positive experiences include the ability to decide a role or which binary (male/female) team to join; students desire mixed groups without binary gender divisions.
Caudwell, 2014	Interviews: Experiences of school sport, sport participation	Transgender, university-aged men (ages 16 to 23)	*n =* 2	Being out as transgender during the transition process caused one participant to quit sport. Being out and transgender creates complications both the field and in the locker room.
Caudwell, 2022	Interviews, focus groups, and drawings were used to document experiences with physical activity/recreational swimming	Members of a transgender social group that participates in monthly recreational swimming in the United Kingdom (age 18+)	Interviews *n =* 9 Focus groups *n =* 3 Observation of group gatherings *n =* 9 Attending swim sessions *n =* 11 Participant ‘drawings’ *n =* 63	Swimming has a positive effect on transgender people’s well-being. Swimming/pools are often unwelcoming spaces to TGD people. In trans-inclusive swim sessions, participants feel free from bodily scrutiny normally seen in spaces such as locker rooms.
Elling-Machartzki 2017	Interviews: Body self-narratives and role of physical activity/sport	Transgender people in the Netherlands (ages 27–51)	*n =* 12	Before transition participants described feelings of alienation and exclusion in PE, PA, and sport. During transition they identified dangerous and safe PA/sport spaces. More of the respondents participated in individual fitness activities than group sport
Hargie et al., 2017	Not measured	Transgender members of a support group in Northern Ireland (ages 25–62)	*n =* 10	Participants felt alienated from sport and gender-activity due to binary gender separation. Participants experience wide-ranging stress due to exclusion; fear, perceived obstacles, and negative past experiences informed participants’ choice to not engage in sport or physical activity.
Herrick & Duncan, 2018	Self-reported athlete status (past/current experiences with sport)	Self-identifying lesbian, gay, bisexual, transgender, queer (LGBTQ+) participants in four large Canadian cities (ages 18+, mean age = 28)	Total *n =* 42 TGD *n =* 11	Most participants do not identify with the term “athlete.” Overlapping intersectionality creates complex experiences when cis-sexism is perpetuated in sport. Sport and physical education are unsafe due to homo/queer-phobia; locker rooms are traumatic spaces.
Herrick et al. 2022	Recommendations for increased inclusion in physical activity	LGBTQ+ adults in North America (ages 18–67, mean age = 30.1, SD = 9.1)	Total *n =* 766 TGD *n =* 228	Inclusionary practices identified include: LGBTQ+ memberships, training for facility staff, advertising inclusion, supportive policies on antidiscrimination, diverse representation, single stalls/gender-neutral changing rooms, and using other parameters such as skill level instead of binary gender divisions in PA.
Jones et al., 2017	Factors associated with physical activity/sport	Young adult patients of a transgender health service in the United Kingdom (ages 18–36, mean age = 22.71)	*n =* 14	Barriers included binary locker rooms and lack of private spaces to change and shower; participants desire more gender-neutral sportswear and mixed gender sports teams. Facilitators included motivation for increased body satisfaction/confidence, body change, and access to trans-only or trans-safe spaces.
Lopez-Canada et al., 2021	Interviews: Participation in physical activity/sport	Spanish transgender people (ages 15–62, mean = 32.76)	*n =* 43	Body appearance, or ‘passing,’ and fear of being outed affects participation in PA/sport. Participants rely on PA/sport to manage hormone treatment side effects or to accelerate desired effects of the drugs. Social support is essential for PA/sport participation. Past experiences with physical education influence attitudes about PA/sport. Gyms, locker rooms, and swimming pools were considered particularly unsafe.
Lucas-Carr & Krane, 2012	Interviews: Self-identifying athlete, sport history	Self-identifying trans athletes (ages 23, 28, and 29)	*n =* 3	Three themes were identified: sport and binary sex segregation, safe spaces for participation in sport, and transgender inclusion within sport.
Phipps, 2021	Focus groups: Self-reported sport participation	Student union officers (ages 18+) within universities in England alongside one transgender student (age 20 years)	Total *n =* 8 TGD = 1	Gender-typing in sport restricts activities to social constructions of gender, which may discourage participation. Sport operates within the gender binary; even in mixed-gender sports, gender still matters.
Serrano et al., 2019	Self-report of physical activities and it’s correlation to body shaping	Transgender women who receive care at a transgender clinic in Brazil (ages 22–45)	*n =* 10	Participants report engaging in a variety of physical activity for either increasing muscle mass in the gluteus, legs, or abdominals. Those who do not exercise were either fearful of masculinizing their bodies or experiencing prejudice.
Storr et al., 2022	Semi-structured interviews: Attitudes to and experiences of sport/PA	Sexuality and gender diverse (SAGD) people who participate in a support service for young people in Australia (ages 18–24)	*n =* 13	SAGD have a strong desire to engage in sport/PA; Sport is considered an exclusionary environment with both explicit and subtle bullying.
Teti et al., 2020	Semi-structured interviews related to body image and exercise priorities	Trans masculine young adults in the Midwest United States (ages 19–25)	*n =* 16	Exercise and body image are connected. Themes emerged include body shape as a motivation to exercise, poor body image, barriers to exercise including stigma and fear, and exercise (or lack of) is destructive. Gyms are unwelcoming.
Zanin et al., 2023	Interviews: Gender identity negation in sport experiences	Transgender and gender non-conforming (TGNC) athletes (ages 24–50, mean age = 30.75, SD = 6.7)	*n =* 20	Themes revealed in the participants’ stories include: gender sanctioning, binary gender survival, transition and disclosure, and stories of gender affirmation; master narratives within sport that produce binary structures and exclusion; counter narratives that challenge the binary sport structures.

### Sport in the male/female binary

Themes of the male/female binary used in sport to divide teams was explored in narratives in students by Lucas-Carr & Krane [28], Phipps [29], and Zanin et al. [30]. Gender-typing in sports discourages sport participation for TGD individuals [28, 29]. One participant in a study by Phipps stated, “Without a trans* identity that ‘fits’ into the binary system, it may be exceptionally difficult to participate in university sport, unless that identity is compromised in an attempt to conform” [29, p. 10]. Youth emphasized the need for more mixed-gender teams to be inclusive of transgender students [29].

Zanin et al. [30] discussed themes of gender sanctioning and binary gender survival. A trans masculine student who was assigned female gender at birth was interviewed; the term trans masculine is a term to identify masculine trans identities that takes into consideration a broad range of identities [4]. This transmasculine basketball athlete speaks about navigating the stereotypical expectations of behavior for feminine athletes (even though the student presents as male) to not play aggressively [30]. The student recounted being punished after fouling a member of the opposite team, “That’s what you’re supposed to do in basketball. But because I was a girl that was considered uncouth. So he sat me down the whole rest of the game as punishment for that I had done” [30, p. 10] Hargie et al. [6] advocated for softening the gender binary in sports after participants in their study report feelings of alienation from sport due to binary gender separation.

### Body image

Body image can be a complicating or facilitating factor in participation in PA for TGD people. Teti et al. [31] found that exercise and body image are interconnected for transgender individuals. Body shape, not overall health, was a motivation to exercise for the transmasculine young adults he interviewed. These participants also reported poor body image as a barrier to exercise. Jones et al. [32] revealed body dissatisfaction as a barrier to PA and sport as well as body satisfaction as a facilitator to PA and sport. One transgender male stated, “The dysphoria is a motivator to change it, it’s not necessarily particularly comfortable but it is a motivator to change it into something that’s more desirable” [32, p. 18]. Berg and Kokkonen [5] described problematic relationships with the body, which made some activities and clothing, such as t-shirts, uncomfortable. They also reported the use of chest-binders as a negative factor associated with activity due to the increased difficulty in breathing caused by the devices.

In Serrano et al. [33] transgender women in Brazil report engaging in a variety of physical activities with the goal of increasing muscle mass in their gluteus, legs, or abdominals. Body image was a barrier for those who did not exercise, citing fear of masculinizing their bodies in addition to the risk of experiencing prejudice. In Elling-Machartzi’s study [34], transgender participants felt excluded in physical education, PA, and sport before transition. During transition they identified both dangerous and safe spaces for physical activity. These transgender participants more often participated in individual fitness activities than group sports. Findings demonstrate that sports and physical activities do not have to be viewed as punishing, dangerous, or shameful for transgender individuals. In fact, when conducted in a supportive social environment, these activities can be enjoyable and empowering, while also contributing to the development of body awareness and gender identification throughout different stages of transition.

Body appearance or the ability to “pass” in their gender and the fear of being outed affects participation in PA and sport among Spanish transgender people [8]. The participants also report using PA to manage hormone treatment side effects or accelerating the effects of these medications. Hormones, which help align the body to the person’s gender, were also a source of fear as one participant noted, “I wanted to compete, but if I’m taking hormones, I cannot compete any more… If I’m taking hormones I cannot compete as a man because my sport level in the men category is poor and I cannot compete as a woman neither. I’m then in a limbo” [8, p. 68]. In Teti et al. [31] a transmasculine young adult stated, “[I want to] look more masculine like the guys lifting tons of weights. Building muscle makes me feel better in general. And the more exercise, depending on the area, you can change, you can have big arms… If you have big arm muscles, it looks very masculine… The more work you do with arms and shoulders, it can help reduce chest size, which is super [important to reduce breast size pre-surgery]” [31, p. 213].

### Exclusion and discrimination

Being excluded or discriminated against was a recurring theme for the participants in these studies. Half of the Finnish students interviewed by Berg and Kokkonen [5] reported witnessing discrimination in physical education classes. The students had varied experiences with discrimination that ranged from no experience to physical harassment. Another study interviewed LGBTQ+ individuals to better understand the complex experiences with PA within this population [7]. Most participants did not identify with the word “athlete.” Intersecting minority identities and cis sexism was a theme that is perpetuated through sport. Negative experiences in sport and physical education informed participants’ lack of identification with being an “athlete” even though they were physically active.

Transgender participants reported fear, perceived obstacles, and negative past experiences kept them from engaging in PA and sport in a study by Hargie et al. [6]. Participants from Australia considered sport to be exclusionary with both explicit and subtle bullying [35]. One university-aged transgender man stated being out as a transgender person who was in the transition process caused him to quit sport [36]. Additionally, Caudwell’s study indicated LGBT+ sports leagues may not be as welcoming to transgender members as they are to lesbian and gay members.

### Exclusionary spaces

The theme of exclusion was further demonstrated by the structures and environments in which PA and sport takes place. In addition to the binary nature of teams and group sport, locker or changing rooms, gyms, and pools were considered particularly upsetting spaces for transgender people. Spanish transgender people describe gyms, locker rooms, and swimming pools as particularly unsafe [8]. A 20-year-old transgender man stated, “No, I never go to gyms. Before chest surgery, I was too anxious. And now [after surgery], I rather prefer to exercise alone at home. I do not feel comfortable with many people and so […] that’s why I have the gym at home” [8, p. 72]. Gyms were also seen as unwelcoming by the transmasculine participants in Teti et al.’s interviews [31].

Locker rooms were deemed traumatic, and sport or physical education were considered unsafe by Canadian participants [7]. Out transgender men at university stated being out and transgender created complications for them both on the field and in the locker room [36]. Negative locker room experiences were also reported by Berg and Kokkonen [5] and Jones et al. [32]. Lack of private spaces to change or shower and binary locker rooms were considered barriers to PA and sport by young adult transgender people in the UK [32]. Single stalls or gender-neutral changing rooms were considered inclusionary practices in Herrick et al.’s study of LGBTQ+ adults in North America, of which 228 participants were transgender or gender diverse [37].

Swimming pools were seen as unwelcoming spaces in members of a UK-based transgender social group that participates in month recreational swimming [38]. Although swimming had a positive effect on well-being, transgender people found the sport and pools unwelcoming. In trans-inclusive swim sessions, the participants felt free from the bodily scrutiny they normally felt in these spaces, which included locker rooms.

### Inclusionary practices

Affirming spaces, supportive peers, representation, mixed gender teams, gender-neutral locker rooms, social support, and supportive policies contribute to inclusion and participation in physical activity and sport [5, 8, 30, 37, 39]. A common theme in supporting women and gender diverse athletes is positive representation and community leadership [39]. Feeling represented and valued was tied to the ability to be engaged in the group [39]. Women, trans, and femme rides and mechanic workshops are two examples of ways participants felt valued in their identity and supported to participate in cycling [39].

Finnish students describe positive experiences when able to decide a role or which team (male/female) to join [5]. These students also desire mixed gender teams devoid of binary divisions. Access to trans-only or trans-safe spaces were facilitators for participation in physical activity and sport [32]. The availability of LGBTQ+ memberships to athletic facilities, training for facility staff, advertising inclusion, supportive policies, gender-neutral changing rooms, and using skill-level rather than gender division in teams were identified as inclusionary practices [37].

## Discussion

This systematic literature review aimed to explore the experiences of transgender and gender diverse individuals in PA and sport, with a focus on policy, systems, and environmental implications. The review included 31 studies, comprising 14 cross-sectional, one cohort, and 16 qualitative studies. The results suggest that transgender individuals have lower rates of PA compared to cisgender or sexual minority individuals [14–16, 18–22, 24, 25]. Trans women were less likely to engage in PA than trans men [21, 25]. Additional evidence to understand PA for transgender individuals is needed. Prevalence studies recommend ongoing surveillance that collects data on transgender individuals, as well as analyzing data separately for trans women and other gender and sexual minorities.

Specific topics that need additional study include disparities in PA and health outcomes by gender identity, understanding facilitators of PA and sport for transgender individuals, experiences of bullying, safety, and inclusion. Examination of transgender narratives related to PA reveals several implications for policy, advocacy, and future research. First, the stories of the transgender people who participated in these studies indicate a need for “softening” the gender binary in sport [6]. Additional, implementation of mixed-gender or trans-inclusive sport and gender-neutral, private, or trans-only spaces such as locker rooms will promote inclusion [5–7, 32, 37, 38].

Body image was found to be a complicating or facilitating factor in participation in PA for transgender individuals. Poor body image was identified as a barrier to exercise, while body satisfaction was a facilitator. Body shape, rather than overall health, may be a powerful motivator to exercise for transgender individuals [31]. Transgender individuals may have a complex relationship with their bodies, which make some activities and clothing, such as t-shirts, uncomfortable. Hormone therapy, which helps align the body to the person’s gender, was also a source of fear and a factor affecting participation, particularly sport [8]. Recent controversies in high level sport warrant additional research on this topic [40].

Exclusion and discrimination were also identified as major themes in the studies. Locker rooms and bathroom are a particularly challenging places for transgender youth and college-aged students and adults where fear, discrimination, and violence may occur. Institutions should consider creating private spaces to change and shower so that all people are comfortable, including transgender individuals. Within programming, gender binary sports and PE classes are viewed as exclusionary. Softening the gender binary in sports may also improve inclusion and engagement in PA for transgender individuals. Developing policy that supports PA for transgender individuals, including for youth and older transgender adults is needed. Anti-discrimination, anti-harassment, and supportive transgender policies should be enacted in PA spaces at all levels [28, 37, 39].

PA may vary before, during, and after gender transition. In Elling-Machartzi’s study, participants felt excluded in physical education, PA, and sport before their transition [34]. During transition, they identified both dangerous and safe spaces to engage in PA. Participants reported using PA to manage hormone treatment side effects or accelerate the effects of these medications. After transition, participants felt liberated to show their bodies and go to gyms and participate in sports that they had quit during transition. It appears transitioning may be a positive aspect for participation in PA and sport.

The findings of this systematic literature review have important policy, systems, and environmental implications for increasing inclusion and transgender-affirming PA and sport experiences. The gender binary structure of sport must be softened to make it more inclusive of transgender individuals. Mixed-gender teams should be introduced to create a more inclusive environment. Policies and guidelines should be established to promote the inclusion of transgender individuals in sport, and training should be provided to coaches and staff to ensure that they are aware of the needs and experiences of transgender athletes. In addition, there is a need for the development of gender-affirming PA and sport programs that are specifically designed for transgender individuals. These programs should be developed by transgender individuals to ensure that they are inclusive and respectful of their needs and experiences.

Interventions across the social ecological levels need to be developed to increase PA for transgender individuals. Specifically, interventions need to improve efforts to develop and maintain inclusive environments for PA and sport. To create affirming spaces advocacy for inclusivity is needed to create equitable opportunities for transgender athletes [39]. Positive steps include awareness campaigns for public education, increased transgender inclusion in physical education programs, gender-awareness training of physical education teachers, and action by those who work in PA as they have the potential to promote inclusion[5, 6, 8, 32, 38].

Limitations of this systematic literature review include the limited number of studies available and the lack of standardization in the measurement of PA and sport participation among transgender individuals. We include only studies that report PA for transgender individuals and we exclude studies that group LGBT+ individuals together to report physical activity. We feel that this is necessary, as Smalley, et al. [25] report that transgender individuals engage in less physical activity than their gay or lesbian peers. However, we may have excluded studies that provide insight into LGBT+ populations. Many of the studies reviewed were qualitative in nature, limiting the generalizability of the findings. The studies were also conducted in different countries with different cultural and social contexts. Generalizing the results should be limited, as context matters for the health of transgender populations. Moreover, most of the studies included in this review focused on binary transgender individuals, leaving out non-binary and gender non-conforming individuals. Additionally, due to the sensitive nature of the topic, it is possible that some transgender individuals may have been reluctant to participate in research or may have provided socially desirable responses, which could have affected the accuracy of the data collected. Furthermore, the majority of the studies focused on the experiences of transgender individuals in sport and PA, rather than on their health outcomes. Therefore, more research is needed to fully understand the relationship between PA and health outcomes in this population.

Recently, several state legislatures in the United States have banned transgender youth in sport participation and require youth to use locker rooms and bathrooms according to their gender assigned at birth [41–43]. These broad state-level polices likely have a direct impact on the PA of transgender individuals, particularly transgender girls. In general, healthcare providers oppose these policies [41, 42, 44]. These same transgender girls are less likely to be active than transgender boys or their cisgender peers. These policies may also increase gender-based discrimination and violence in PA settings. Policy aimed at limiting sport and PA participation among transgender individuals may also have long-term impacts on the mental and physical health of an already marginalized transgender population.

This systematic literature review provides some insights into the experiences of transgender individuals in sport and PA. However, more research is needed to understand the complex interactions between gender identity, PA, and health outcomes. Standardization of measurement and inclusion of diverse transgender populations are essential to improve findings and to better inform policies and interventions aimed at promoting PA and health equity among transgender individuals. Future interventions and policy should aim to increase PA for transgender populations. This research should be codesigned and coproduced with members of the transgender community, including transgender researchers and community members.

## Supporting information

S1 Checklist PRISMA 2020 checklist. (DOCX)
